# Undeca­carbonyl-μ_2_-methane­thiol­ato-μ_2_-[(pyridin-2-yl)methane­thiol­ato]-μ_4_-sulfido-tetra­iron(II)(2 *Fe*—*Fe*)

**DOI:** 10.1107/S1600536811047751

**Published:** 2011-11-16

**Authors:** Yao-Cheng Shi, Liang Lai, Wen-Bin Shen, Li-Min Yuan

**Affiliations:** a College of Chemistry and Chemical Engineering, Yangzhou University, Yangzhou 225002, People’s Republic of China; b Analytical Center, China Pharmaceutical University, Nanjin 210009, People’s Republic of China; c Testing Center, Yangzhou University, Yangzhou 225009, People’s Republic of China

## Abstract

The title compound, [Fe_4_(C_6_H_6_NS)(CH_3_S)S(CO)_11_], com­prises two butterfly-shaped sub-cluster cores, Fe_2_S_2_N and Fe_2_S_2_, joined together by a spiro-type μ_4_-S atom. The (pyridin-2-yl)methane­thiol­ate ligand is attached to the Fe_2_(CO)_5_ unit in a μ-κ*N*:κ^2^ *S* mode, and the methane­thiol­ate ligand is coordinated to the Fe_2_(CO)_6_ unit in a μ-κ^2^ *S* fashion.

## Related literature

For general background to iron–carbonyl clusters, see: Capon *et al.* (2009 ▶); Tard & Pickett (2009 ▶); Gloaguen & Rauchfuss (2009 ▶); DuBois & DuBois (2009 ▶). For the syntheses of μ_4_-S atom-containing Fe_2_(CO)_6_ butterfly-shaped complexes, see: Song (2005 ▶); Wang *et al.* (2000 ▶). For related structures, see: Song *et al.* (2000 ▶, 2002 ▶).
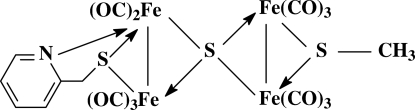


## Experimental

### 

#### Crystal data

[Fe_4_(C_6_H_6_NS)(CH_3_S)S(CO)_11_]
*M* *_r_* = 734.87
Monoclinic, 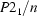

*a* = 9.1253 (3) Å
*b* = 28.9515 (15) Å
*c* = 10.0376 (11) Å
β = 98.3238 (12)°
*V* = 2623.9 (3) Å^3^
*Z* = 4
Mo *K*α radiation
μ = 2.46 mm^−1^
*T* = 296 K
0.19 × 0.16 × 0.15 mm

#### Data collection

Bruker SMART APEX CCD diffractometer
Absorption correction: multi-scan (*SADABS*; Sheldrick, 2004 ▶) *T*_min_ = 0.628, *T*_max_ = 0.684
22650 measured reflections
6151 independent reflections
4914 reflections with *I* > 2σ(*I*)
*R*_int_ = 0.032

#### Refinement

*R*[*F* ^2^ > 2σ(*F* ^2^)] = 0.049
*wR*(*F* ^2^) = 0.093
*S* = 1.19
6151 reflections
335 parameters
H-atom parameters constrained
Δρ_max_ = 0.45 e Å^−3^
Δρ_min_ = −0.50 e Å^−3^

### 

Data collection: *SMART* (Bruker, 2002 ▶); cell refinement: *SAINT-Plus* (Bruker, 2003 ▶); data reduction: *SAINT-Plus*; program(s) used to solve structure: *SIR2004* (Burla *et al.*, 2005 ▶); program(s) used to refine structure: *SHELXTL* (Sheldrick, 2008 ▶); molecular graphics: *PLATON* (Spek, 2009 ▶) and *WinGX* (Farrugia, 1999 ▶); software used to prepare material for publication: *publCIF* (Westrip, 2010 ▶).

## Supplementary Material

Crystal structure: contains datablock(s) I, global. DOI: 10.1107/S1600536811047751/tk5011sup1.cif

Structure factors: contains datablock(s) I. DOI: 10.1107/S1600536811047751/tk5011Isup2.hkl

Additional supplementary materials:  crystallographic information; 3D view; checkCIF report

## Figures and Tables

**Table 1:** Selected bond lengths (Å)

Fe1—Fe2	2.5394 (9)
Fe1—S1	2.2968 (14)
Fe1—S2	2.2525 (11)
Fe2—S1	2.2401 (13)
Fe2—N1	2.022 (3)
Fe2—S2	2.2148 (11)
Fe3—Fe4	2.5473 (9)
Fe3—S2	2.2485 (12)
Fe3—S3	2.2801 (13)
Fe4—S2	2.2428 (11)
Fe4—S3	2.2761 (13)

**Fig. 1.**
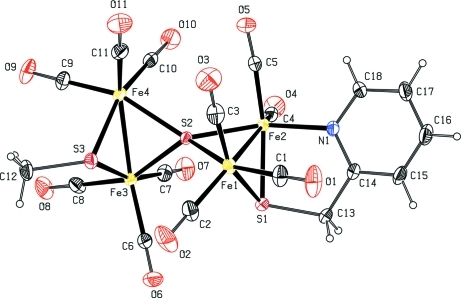
The molecule of the title compound, showing the atom-labelling scheme. Displacement ellipsoids are drawn at the 20% probability level.

## supplementary crystallographic information

### Comment

Recently, Fe/S cluster complexes have attracted considerable attention, because of their interesting chemistry and particularly their close relevance to the modeling study of the active site of [Fe—Fe] hydrogenases. Moreover, until now, few efficient electrocatalysts have been obtained and the mechanism of the natural production/uptake of hydrogen remains unclear. Therefore, novel structural and chemical models are still necessary to gain a better understanding of the protonation mechanisms implied at the molecular level (Capon *et al.*, 2009; Tard & Pickett, 2009; Gloaguen & Rauchfuss, 2009; DuBois & DuBois, 2009). The reaction sequence 2–C_5_H_4_NCH_2_SH/Fe_3_(CO)_12_/Et_3_N/CS_2_/MeI in THF leads to the formation of the title compound (Song, 2005; Wang *et al.*, 2000). Its molecular structure consists of the two butterfly sub-cluster cores Fe1Fe2S1N1S2 and Fe3Fe4S2S3 joined together to a spiro type of µ_4_-S atom, *i.e.*, S2 (Fig. 1 and Table 1). The ligand 2–C_5_H_4_NCH_2_S^-^ is attached to Fe_2_(CO)_5_ unit in a µ–*kN*:*k*^2^*S* mode while the ligand CH_3_S^-^ is coordinated to Fe_2_(CO)_6_ unit in a µ–*k*^2^*S* fashion. Interestingly, as seen from Table 1, the S3 atom is symmetrically coordinated to the Fe3—Fe4 bond while the S1 atom is asymmetrically to the Fe1—Fe2 bond. As in the related complex (µ–MeS)Fe_2_(CO)_6_(µ_4_–S)Fe_2_(CO)_6_(µ–SCSMe) (Song *et al.*, 2000, 2002), the CH_3_ group is bonded to the S3 atom by an equatorial type of bond. The IR spectrum displays four absorption bands due to terminal carbonyl ligands. As expected, because of a chiral butterfly core, its ^1^H NMR spectrum shows for the CH_2_ group an AB quartet characteristic of nonequivalent hydrogen atoms. Also, its ^13^H NMR spectrum exhibits the corresponding absorption peaks which are in agreement with the aforementioned X-ray diffraction analysis.

### Experimental

A solution of Fe_3_(CO)_12_ (1.00 g, 2 mmol) and 2–pyridinemethanethiol (0.25 g, 2 mmol) in 15 mL of THF was stirred under inert atmosphere for 30 min. Then CS_2_ (0.30 g, 4 mmol) was added and the solution stirred for 5 h. The solution was cooled to 0 °C and MeI (0.57 g, 4 mmol) added. After being stirred overnight at room temperature, the solvent was removed and the resulting residue was purified by chromatography on silica gel with petroleum ether as eluant to give the brown-red solid. Single crystals were grown from its dichloromethane-petroleum ether solution. IR (KBr): ν(C≡O) 2071 (*m*), 2030 (*vs*), 1997 (*s*), 1952 (*s*) cm^-1^. ^1^H NMR (500 MHz, CDCl_3_, δ, p.p.m.): 8.71, 7.48-6.98 (s, 1H, m, 3H, C_5_H_4_N), 4.26, 3.93 (AB quartet, ^2^*J* = 15 Hz, 1H, 1H, CH_2_), 2.10 (s, 3H, CH_3_). ^13^C NMR (125 MHz, CDCl_3_, δ, p.p.m.): 21.6 (CH_3_), 43.4 (CH_2_), 122.9, 136.1, 155.5, 166.1 (C_5_H_4_N), 207.9, 208.2, 210.8, 211.6, 213.4, 216.1 (C≡O).

### Refinement

The H atoms were geometrically placed (C—H = 0.93–0.97 Å) and refined as riding with *U_iso_*(H) = 1.2*U_eq_*(C) and *U_iso_*(H) = 1.5*U_eq_*(methyl-C).

### Crystal data

**Table:**

[Fe_4_(C_6_H_6_NS)(CH_3_S)S(CO)_11_]	*F*(000) = 1456
*M**_r_* = 734.87	*D*_x_ = 1.860 Mg m^−^^3^
Monoclinic, *P*2_1_/*n*	Mo *K*α radiation, λ = 0.71073 Å
Hall symbol: -P 2yn	Cell parameters from 4914 reflections
*a* = 9.1253 (3) Å	θ = 2.2–27.9°
*b* = 28.9515 (15) Å	µ = 2.46 mm^−^^1^
*c* = 10.0376 (11) Å	*T* = 296 K
β = 98.3238 (12)°	Block, red
*V* = 2623.9 (3) Å^3^	0.19 × 0.16 × 0.15 mm
*Z* = 4	

### Data collection

**Table:**

Bruker SMART APEX CCD diffractometer	6151 independent reflections
Radiation source: fine-focus sealed tube	4914 reflections with *I* > 2σ(*I*)
graphite	*R*_int_ = 0.032
ω and φ scans	θ_max_ = 27.8°, θ_min_ = 2.2°
Absorption correction: multi-scan (*SADABS*; Sheldrick, 2004)	*h* = −11→11
*T*_min_ = 0.628, *T*_max_ = 0.684	*k* = −36→37
22650 measured reflections	*l* = −12→13

### Refinement

**Table:**

Refinement on *F*^2^	Primary atom site location: structure-invariant direct methods
Least-squares matrix: full	Secondary atom site location: difference Fourier map
*R*[*F*^2^ > 2σ(*F*^2^)] = 0.049	Hydrogen site location: inferred from neighbouring sites
*wR*(*F*^2^) = 0.093	H-atom parameters constrained
*S* = 1.19	*w* = 1/[σ^2^(*F*_o_^2^) + (0.*P*)^2^ + 7.4281*P*] where *P* = (*F*_o_^2^ + 2*F*_c_^2^)/3
6151 reflections	(Δ/σ)_max_ < 0.001
335 parameters	Δρ_max_ = 0.45 e Å^−^^3^
0 restraints	Δρ_min_ = −0.50 e Å^−^^3^

### Special details

**Table:**

Geometry. All e.s.d.'s (except the e.s.d. in the dihedral angle between two l.s. planes) are estimated using the full covariance matrix. The cell e.s.d.'s are taken into account individually in the estimation of e.s.d.'s in distances, angles and torsion angles; correlations between e.s.d.'s in cell parameters are only used when they are defined by crystal symmetry. An approximate (isotropic) treatment of cell e.s.d.'s is used for estimating e.s.d.'s involving l.s. planes.
Refinement. Refinement of *F*^2^ against ALL reflections. The weighted *R*-factor *wR* and goodness of fit *S* are based on *F*^2^, conventional *R*-factors *R* are based on *F*, with *F* set to zero for negative *F*^2^. The threshold expression of *F*^2^ > σ(*F*^2^) is used only for calculating *R*-factors(gt) *etc*. and is not relevant to the choice of reflections for refinement. *R*-factors based on *F*^2^ are statistically about twice as large as those based on *F*, and *R*- factors based on ALL data will be even larger.

### Fractional atomic coordinates and isotropic or equivalent isotropic displacement parameters (Å^2^)

**Table:**

	*x*	*y*	*z*	*U*_iso_*/*U*_eq_	
C1	0.3087 (6)	0.1314 (2)	−0.0125 (5)	0.0612 (15)	
C2	0.1355 (7)	0.1881 (2)	0.1127 (5)	0.0654 (16)	
C3	0.1362 (6)	0.0911 (2)	0.1218 (5)	0.0624 (15)	
C4	0.6043 (5)	0.09347 (17)	0.4471 (5)	0.0459 (11)	
C5	0.3874 (5)	0.04298 (17)	0.3159 (5)	0.0454 (11)	
C6	0.2725 (5)	0.23874 (17)	0.4290 (5)	0.0473 (11)	
C7	0.4244 (6)	0.17741 (18)	0.6064 (5)	0.0492 (12)	
C8	0.1887 (6)	0.20957 (17)	0.6739 (5)	0.0522 (12)	
C9	0.0277 (5)	0.1125 (2)	0.6643 (5)	0.0543 (13)	
C10	0.2578 (6)	0.07232 (18)	0.5986 (5)	0.0492 (12)	
C11	−0.0013 (6)	0.06344 (19)	0.4274 (6)	0.0553 (13)	
C12	−0.1349 (6)	0.1994 (2)	0.5184 (6)	0.0664 (16)	
H12A	−0.2317	0.1881	0.4837	0.100*	
H12B	−0.1330	0.2324	0.5089	0.100*	
H12C	−0.1116	0.1914	0.6119	0.100*	
C13	0.6172 (6)	0.16550 (17)	0.1184 (6)	0.0591 (15)	
H13A	0.5847	0.1828	0.0368	0.071*	
H13B	0.7106	0.1785	0.1601	0.071*	
C14	0.6407 (5)	0.11606 (17)	0.0822 (5)	0.0463 (11)	
C15	0.7145 (6)	0.1058 (2)	−0.0263 (5)	0.0627 (15)	
H15	0.7458	0.1294	−0.0782	0.075*	
C16	0.7408 (6)	0.0605 (2)	−0.0560 (6)	0.0666 (16)	
H16	0.7893	0.0530	−0.1284	0.080*	
C17	0.6937 (6)	0.0267 (2)	0.0236 (6)	0.0659 (16)	
H17	0.7109	−0.0043	0.0062	0.079*	
C18	0.6214 (5)	0.03872 (17)	0.1287 (5)	0.0510 (12)	
H18	0.5920	0.0153	0.1823	0.061*	
Fe1	0.25779 (7)	0.13887 (2)	0.14905 (6)	0.04061 (16)	
Fe2	0.46539 (6)	0.09889 (2)	0.30222 (6)	0.03290 (14)	
Fe3	0.24023 (7)	0.18660 (2)	0.52126 (6)	0.03460 (15)	
Fe4	0.11363 (7)	0.10806 (2)	0.51490 (6)	0.03607 (15)	
N1	0.5907 (4)	0.08240 (13)	0.1587 (4)	0.0406 (9)	
O1	0.3419 (5)	0.1255 (2)	−0.1178 (4)	0.1053 (19)	
O2	0.0599 (6)	0.21896 (19)	0.0902 (5)	0.114 (2)	
O3	0.0630 (6)	0.05878 (19)	0.1038 (5)	0.1026 (17)	
O4	0.6892 (4)	0.08923 (15)	0.5427 (4)	0.0741 (12)	
O5	0.3306 (5)	0.00851 (13)	0.3287 (5)	0.0763 (12)	
O6	0.2891 (5)	0.27134 (13)	0.3701 (4)	0.0717 (12)	
O7	0.5400 (4)	0.17148 (16)	0.6638 (4)	0.0743 (12)	
O8	0.1601 (5)	0.22297 (15)	0.7736 (4)	0.0820 (13)	
O9	−0.0211 (5)	0.11492 (18)	0.7626 (4)	0.0858 (14)	
O10	0.3524 (5)	0.05028 (16)	0.6518 (4)	0.0795 (13)	
O11	−0.0745 (5)	0.03477 (16)	0.3740 (5)	0.0909 (15)	
S1	0.48087 (14)	0.17202 (4)	0.23270 (12)	0.0434 (3)	
S2	0.26921 (11)	0.13019 (3)	0.37332 (9)	0.0301 (2)	
S3	0.00216 (12)	0.17347 (4)	0.42431 (12)	0.0420 (3)	

### Atomic displacement parameters (Å^2^)

**Table:**

	*U*^11^	*U*^22^	*U*^33^	*U*^12^	*U*^13^	*U*^23^
C1	0.048 (3)	0.090 (4)	0.045 (3)	0.015 (3)	0.005 (2)	0.003 (3)
C2	0.072 (4)	0.078 (4)	0.046 (3)	0.029 (3)	0.011 (3)	0.010 (3)
C3	0.056 (3)	0.082 (4)	0.046 (3)	0.003 (3)	−0.002 (2)	−0.012 (3)
C4	0.038 (2)	0.048 (3)	0.054 (3)	0.002 (2)	0.014 (2)	0.006 (2)
C5	0.046 (3)	0.042 (3)	0.052 (3)	0.006 (2)	0.017 (2)	−0.002 (2)
C6	0.051 (3)	0.044 (3)	0.049 (3)	0.000 (2)	0.015 (2)	−0.005 (2)
C7	0.046 (3)	0.055 (3)	0.047 (3)	−0.008 (2)	0.009 (2)	−0.010 (2)
C8	0.066 (3)	0.043 (3)	0.047 (3)	0.009 (2)	0.007 (2)	−0.004 (2)
C9	0.043 (3)	0.071 (4)	0.051 (3)	0.004 (3)	0.016 (2)	0.008 (3)
C10	0.051 (3)	0.051 (3)	0.049 (3)	0.000 (2)	0.019 (2)	0.011 (2)
C11	0.049 (3)	0.057 (3)	0.060 (3)	−0.005 (3)	0.009 (2)	0.007 (3)
C12	0.046 (3)	0.077 (4)	0.082 (4)	0.021 (3)	0.026 (3)	0.002 (3)
C13	0.070 (4)	0.046 (3)	0.073 (4)	0.000 (3)	0.048 (3)	0.009 (3)
C14	0.041 (2)	0.052 (3)	0.050 (3)	−0.002 (2)	0.020 (2)	0.001 (2)
C15	0.068 (4)	0.072 (4)	0.057 (3)	−0.010 (3)	0.040 (3)	−0.002 (3)
C16	0.064 (4)	0.084 (4)	0.060 (3)	−0.011 (3)	0.035 (3)	−0.017 (3)
C17	0.060 (3)	0.061 (4)	0.084 (4)	0.002 (3)	0.035 (3)	−0.023 (3)
C18	0.050 (3)	0.045 (3)	0.062 (3)	0.012 (2)	0.020 (2)	0.002 (2)
Fe1	0.0455 (4)	0.0488 (4)	0.0281 (3)	0.0108 (3)	0.0070 (3)	0.0025 (3)
Fe2	0.0319 (3)	0.0332 (3)	0.0354 (3)	0.0025 (3)	0.0108 (2)	0.0028 (2)
Fe3	0.0368 (3)	0.0362 (3)	0.0321 (3)	0.0000 (3)	0.0092 (2)	−0.0026 (3)
Fe4	0.0322 (3)	0.0404 (4)	0.0369 (3)	−0.0024 (3)	0.0095 (2)	0.0050 (3)
N1	0.0370 (19)	0.044 (2)	0.043 (2)	0.0056 (17)	0.0139 (16)	0.0025 (17)
O1	0.092 (3)	0.195 (6)	0.031 (2)	0.030 (4)	0.017 (2)	−0.013 (3)
O2	0.141 (5)	0.116 (4)	0.082 (3)	0.084 (4)	0.005 (3)	0.023 (3)
O3	0.099 (4)	0.106 (4)	0.097 (4)	−0.039 (3)	−0.005 (3)	−0.024 (3)
O4	0.050 (2)	0.095 (3)	0.071 (3)	0.008 (2)	−0.015 (2)	0.015 (2)
O5	0.089 (3)	0.041 (2)	0.106 (3)	−0.012 (2)	0.038 (3)	−0.003 (2)
O6	0.100 (3)	0.048 (2)	0.072 (3)	−0.001 (2)	0.027 (2)	0.014 (2)
O7	0.043 (2)	0.100 (3)	0.074 (3)	−0.002 (2)	−0.0098 (19)	−0.012 (2)
O8	0.118 (4)	0.085 (3)	0.046 (2)	0.024 (3)	0.024 (2)	−0.017 (2)
O9	0.075 (3)	0.133 (4)	0.058 (2)	−0.004 (3)	0.038 (2)	0.008 (3)
O10	0.067 (3)	0.093 (3)	0.077 (3)	0.027 (2)	0.009 (2)	0.041 (2)
O11	0.086 (3)	0.078 (3)	0.103 (4)	−0.035 (3)	−0.005 (3)	−0.014 (3)
S1	0.0511 (7)	0.0349 (6)	0.0489 (7)	−0.0006 (5)	0.0225 (5)	0.0025 (5)
S2	0.0304 (5)	0.0326 (5)	0.0282 (5)	0.0010 (4)	0.0069 (4)	0.0013 (4)
S3	0.0349 (6)	0.0500 (7)	0.0420 (6)	0.0060 (5)	0.0083 (5)	0.0020 (5)

### Geometric parameters (Å, °)

**Table:**

C1—O1	1.153 (6)	C13—C14	1.500 (7)
C1—Fe1	1.764 (5)	C13—S1	1.820 (5)
C2—O2	1.132 (6)	C13—H13A	0.9700
C2—Fe1	1.814 (6)	C13—H13B	0.9700
C3—O3	1.150 (7)	C14—N1	1.360 (6)
C3—Fe1	1.768 (6)	C14—C15	1.393 (6)
C4—O4	1.150 (6)	C15—C16	1.372 (8)
C4—Fe2	1.792 (5)	C15—H15	0.9300
C5—O5	1.140 (6)	C16—C17	1.372 (8)
C5—Fe2	1.781 (5)	C16—H16	0.9300
C6—O6	1.135 (6)	C17—C18	1.368 (7)
C6—Fe3	1.817 (5)	C17—H17	0.9300
C7—O7	1.140 (6)	C18—N1	1.339 (6)
C7—Fe3	1.791 (5)	C18—H18	0.9300
C8—O8	1.138 (6)	Fe1—Fe2	2.5394 (9)
C8—Fe3	1.795 (5)	Fe1—S1	2.2968 (14)
C9—O9	1.142 (6)	Fe1—S2	2.2525 (11)
C9—Fe4	1.794 (5)	Fe2—S1	2.2401 (13)
C10—O10	1.142 (6)	Fe2—N1	2.022 (3)
C10—Fe4	1.785 (5)	Fe2—S2	2.2148 (11)
C11—O11	1.148 (6)	Fe3—Fe4	2.5473 (9)
C11—Fe4	1.809 (6)	Fe3—S2	2.2485 (12)
C12—S3	1.834 (5)	Fe3—S3	2.2801 (13)
C12—H12A	0.9600	Fe4—S2	2.2428 (11)
C12—H12B	0.9600	Fe4—S3	2.2761 (13)
C12—H12C	0.9600		
			
O1—C1—Fe1	178.5 (6)	C4—Fe2—S2	106.51 (15)
O2—C2—Fe1	179.6 (7)	N1—Fe2—S2	153.28 (11)
O3—C3—Fe1	176.7 (6)	C5—Fe2—S1	157.93 (16)
O4—C4—Fe2	177.3 (5)	C4—Fe2—S1	105.45 (16)
O5—C5—Fe2	175.5 (4)	N1—Fe2—S1	86.20 (11)
O6—C6—Fe3	178.3 (5)	S2—Fe2—S1	78.70 (4)
O7—C7—Fe3	178.1 (5)	C5—Fe2—Fe1	100.93 (16)
O8—C8—Fe3	177.2 (5)	C4—Fe2—Fe1	155.39 (16)
O9—C9—Fe4	177.0 (5)	N1—Fe2—Fe1	97.22 (11)
O10—C10—Fe4	178.4 (5)	S2—Fe2—Fe1	56.06 (3)
O11—C11—Fe4	178.7 (5)	S1—Fe2—Fe1	57.03 (4)
S3—C12—H12A	109.5	C7—Fe3—C8	89.5 (2)
S3—C12—H12B	109.5	C7—Fe3—C6	99.0 (2)
H12A—C12—H12B	109.5	C8—Fe3—C6	102.1 (2)
S3—C12—H12C	109.5	C7—Fe3—S2	90.87 (16)
H12A—C12—H12C	109.5	C8—Fe3—S2	154.62 (17)
H12B—C12—H12C	109.5	C6—Fe3—S2	102.91 (15)
C14—C13—S1	112.8 (3)	C7—Fe3—S3	161.59 (17)
C14—C13—H13A	109.0	C8—Fe3—S3	94.30 (18)
S1—C13—H13A	109.0	C6—Fe3—S3	97.79 (16)
C14—C13—H13B	109.0	S2—Fe3—S3	78.05 (4)
S1—C13—H13B	109.0	C7—Fe3—Fe4	105.66 (17)
H13A—C13—H13B	107.8	C8—Fe3—Fe4	100.27 (17)
N1—C14—C15	121.9 (5)	C6—Fe3—Fe4	146.63 (16)
N1—C14—C13	118.4 (4)	S2—Fe3—Fe4	55.34 (3)
C15—C14—C13	119.7 (4)	S3—Fe3—Fe4	55.93 (4)
C16—C15—C14	119.6 (5)	C10—Fe4—C9	91.7 (2)
C16—C15—H15	120.2	C10—Fe4—C11	98.7 (2)
C14—C15—H15	120.2	C9—Fe4—C11	99.4 (2)
C15—C16—C17	118.4 (5)	C10—Fe4—S2	88.43 (15)
C15—C16—H16	120.8	C9—Fe4—S2	154.46 (19)
C17—C16—H16	120.8	C11—Fe4—S2	105.78 (17)
C18—C17—C16	119.5 (5)	C10—Fe4—S3	157.89 (17)
C18—C17—H17	120.2	C9—Fe4—S3	92.88 (18)
C16—C17—H17	120.2	C11—Fe4—S3	101.88 (17)
N1—C18—C17	123.7 (5)	S2—Fe4—S3	78.25 (4)
N1—C18—H18	118.2	C10—Fe4—Fe3	101.82 (17)
C17—C18—H18	118.2	C9—Fe4—Fe3	99.59 (18)
C1—Fe1—C3	90.3 (3)	C11—Fe4—Fe3	151.45 (17)
C1—Fe1—C2	98.5 (2)	S2—Fe4—Fe3	55.55 (3)
C3—Fe1—C2	103.2 (3)	S3—Fe4—Fe3	56.08 (4)
C1—Fe1—S2	157.68 (18)	C18—N1—C14	116.8 (4)
C3—Fe1—S2	90.26 (18)	C18—N1—Fe2	122.8 (3)
C2—Fe1—S2	103.12 (17)	C14—N1—Fe2	120.3 (3)
C1—Fe1—S1	92.86 (19)	C13—S1—Fe2	100.25 (17)
C3—Fe1—S1	152.38 (19)	C13—S1—Fe1	112.1 (2)
C2—Fe1—S1	103.4 (2)	Fe2—S1—Fe1	68.06 (4)
S2—Fe1—S1	76.76 (4)	Fe2—S2—Fe4	134.64 (5)
C1—Fe1—Fe2	103.19 (17)	Fe2—S2—Fe3	133.46 (5)
C3—Fe1—Fe2	97.69 (19)	Fe2—S2—Fe1	69.28 (4)
C2—Fe1—Fe2	149.67 (19)	Fe4—S2—Fe3	69.11 (4)
S2—Fe1—Fe2	54.66 (3)	Fe4—S2—Fe1	136.32 (5)
S1—Fe1—Fe2	54.91 (4)	Fe3—S2—Fe1	125.89 (5)
C5—Fe2—C4	95.8 (2)	C12—S3—Fe4	115.7 (2)
C5—Fe2—N1	96.52 (18)	C12—S3—Fe3	113.0 (2)
C4—Fe2—N1	98.67 (18)	Fe4—S3—Fe3	67.98 (4)
C5—Fe2—S2	89.81 (15)		

## References

[bb1] Bruker (2002). *SMART* Bruker AXS Inc., Madison, Wisconsin, USA.

[bb2] Bruker (2003). *SAINT-Plus* Bruker AXS Inc., Madison, Wisconsin, USA.

[bb3] Burla, M. C., Caliandro, R., Camalli, M., Carrozzini, B., Cascarano, G. L., De Caro, L., Giacovazzo, C., Polidori, G. & Spagna, R. (2005). *J. Appl. Cryst.* **38**, 381–388.

[bb4] Capon, J. F., Gloaguen, F., Pétillon, F. Y., Schollhammer, P. & Talarmin, J. (2009). *Coord. Chem. Rev.* **253**, 1476–1494.

[bb5] DuBois, M. R. & DuBois, D. L. (2009). *Chem. Soc. Rev.* **38**, 62–72.

[bb6] Farrugia, L. J. (1999). *J. Appl. Cryst.* **32**, 837–838.

[bb7] Gloaguen, F. & Rauchfuss, T. B. (2009). *Chem. Soc. Rev.* **38**, 100–108. 10.1039/b801796b PMC3462221 19088969

[bb8] Sheldrick, G. M. (2004). *SADABS* University of Göttingen, Germany.

[bb9] Sheldrick, G. M. (2008). *Acta Cryst.* A**64**, 112–122. 10.1107/S0108767307043930 18156677

[bb10] Song, L.-C. (2005). *Acc. Chem. Res.* **38**, 21–28. 10.1021/ar030004j 15654733

[bb11] Song, L.-C., Fan, H.-T. & Hu, Q.-M. (2002). *J. Am. Chem. Soc.* **124**, 4566–4567. 10.1021/ja020151x 11971696

[bb12] Song, L.-C., Hu, Q.-M., Fan, H.-T., Sun, B.-W., Tang, M.-Y., Chen, Y., Sun, Y., Sun, C.-X. & Wu, Q.-J. (2000). *Organometallics*, **19**, 3909–3915.

[bb13] Spek, A. L. (2009). *Acta Cryst.* D**65**, 148–155. 10.1107/S090744490804362X PMC2631630 19171970

[bb14] Tard, C. & Pickett, C. J. (2009). *Chem. Rev.* **109**, 2245–2274. 10.1021/cr800542q 19438209

[bb15] Wang, Z.-X., Zhao, H., Zhou, Z.-Y. & Zhou, X.-G. (2000). *J. Organomet. Chem.* **599**, 256–260.

[bb16] Westrip, S. P. (2010). *J. Appl. Cryst.* **43**, 920–925.

